# Simultaneous nutrition removal and high-efficiency biomass and lipid accumulation by microalgae using anaerobic digested effluent from cattle manure combined with municipal wastewater

**DOI:** 10.1186/s13068-019-1553-1

**Published:** 2019-09-12

**Authors:** Lin Luo, Hongyu Ren, Xuanyuan Pei, Guojun Xie, Defeng Xing, Yingqi Dai, Nanqi Ren, Bingfeng Liu

**Affiliations:** State Key Laboratory of Urban Water Resource and Environment, School of Environment, Harbin Institute of Technology, Harbin, 150090 China

**Keywords:** Anaerobic digestion effluent, Lipid production, Wastewater treatment, *Scenedesmus*, Nutrition removal

## Abstract

**Background:**

Microalgae as a viable biodiesel feedstock show great potential to approach the challenges of energy shortage and environment pollution, but their economic feasibility was seriously hampered by high production cost. Thus, it is in urgent need to reduce the cost of cultivation and improve the biomass and lipid production of microalgae. In this work, anaerobic digestion effluent from cattle manure combined with municipal wastewater was used as a cost-effective medium for cultivating microalgae and expected to obtain high biomass. The pretreatment of anaerobic digested effluent containing dilution rate, sterilization and nutrient optimization was investigated. Then, initial pH and light intensity for algal growth, lipid production and wastewater purification were optimized in this study.

**Results:**

*Scenedesmus* sp. could grow rapidly in 10% anaerobic digestion effluent from cattle manure combined with secondary sedimentation tank effluent without sterilization. Optimum nutrient additives for higher biomass were as follows: glucose 10 g/L, NaNO_3_ 0.3 g/L, K_2_HPO_4_·3H_2_O 0.01 g/L, MgSO_4_·7H_2_O 0.075 g/L and trace element A5 solution 1 mL/L. Biomass of 4.65 g/L and lipid productivity of 81.90 mg/L/day were achieved during 7-day cultivation accompanying over 90% of COD, NO_3_^−^-N, NH_4_^+^-N, and 79–88% of PO_4_^3−^-P removal with optimized initial pH of 7.0 and light intensity of 5000 l×. The FAME profile in ADEC growth medium consisted in saturated (39.48%) and monounsaturated (60.52%) fatty acids with the 16- to 18-chain-length fatty acids constituting over 98% of total FAME.

**Conclusions:**

This study proves the potential of anaerobic digested effluent combined with municipal wastewater for microalgae culture, and provides an effective avenue for simultaneous microalgal lipid production and treatment of two kinds of wastewater.

## Background

Due to over-reliance on fossil fuels, energy shortage and environmental pollution have become global issues. In this context, the search for alternative, sustainable and green fuels such as biodiesel has become highly pronounced [1–3]. Microalgae are regarded as the most promising feedstock for biodiesel production because of its high photosynthetic efficiency, high algal productivity, favorable carbon capture and strong environmental adaptability [4–6]. However, the high production costs, especially 22–60% of product energy content in cultivation, have seriously impeded the commercialization pace of microalgae biodiesel [7, 8]. It was estimated that if fresh water was not recycled, 3726 kg of water, 0.33 kg of nitrogen and 0.71 kg of phosphate would be required to produce 1 kg of biodiesel using the existing microalgae culture mode and lipid extraction technology [9]. Since water and nutrients are the core elements of microalgal culture and have a serious impact on the overall energy balance, there is an urgent need to seek an alternative and low-cost medium to maintain the long-term sustainability of microalgal culture [10]. Wastewaters derived from municipal, agricultural and industrial activities were suggested to cultivate microalgae, which could enhance the competitiveness of biodiesel production from microalgae, reduce the competition for fresh water, and add the value of treating wastewater itself [11–13]. Anaerobic digestion (AD) is an attractive waste treatment technology for agricultural wastes [14], especially livestock and poultry manure in China. Up to 2014, China owns 103,036 agricultural waste disposal projects, which produced 0.25 billion m^3^ of biogas [15]. With the increase of biogas production, the large quantities of anaerobic digested effluent (ADE) would produce disastrous influence on the surrounding environment.

ADE is traditionally applied to land as a soil amendment, but improper land application practices can lead to many problems such as ammonia volatilization and over-fertilization [16]. High ammonia nitrogen and low C/N ratio of ADE result in poor performance in aerobic or anaerobic treatment [17]. Microalgae have gained much attention for its potential of water reuse, nutrient utilization and biofuel production [18]. ADE is rich in nutrients (e.g., nitrogen and phosphorus) and maintains a suitable nitrogen–phosphorus ratio for microalgal growth [19]. In the microalgal culture system based on ADE, microalgae can help to provide nutrient removal service for wastewater treatment. In addition, lipid-rich microalgae such as *Scenedesmus* sp. and *Chlorella vulgaris* can accumulate lipids and carbohydrates for biofuels production [20, 21]. Thus, the objectives of wastewater treatment and biofuel feedstock production are aligned, at least in terms of maximizing biomass production [22]. Considering both wastewater treatment and lipid production, microalgae cultivated in different culture modes performed differently. For example, microalgae in photoautotrophic mode are more effective in wastewater purification but less in biomass and lipid production, while microalgae in heterotrophic mode provided less effective purification potential, but more lipid accumulation and higher lipid productivity [23]. Microalgae in mixotrophic mode combined the effects of photoautotrophic and heterotrophic conditions, with good purification potential and high growth and lipid accumulation. Therefore, it is of great significance to select suitable culture mode for microalgae culture in wastewater medium system.

However, ADE presents unique challenges for algal cultivation that are not typically encountered with chemically defined media, including potentially high turbidity and concentrations, and competitive microorganisms [24]. Filtration and sterilization are applied in most researches, but they are difficult for large-scale outdoor culture. The simplest pretreatment method is dilution, which can reduce high concentration of ammonium and turbidity of ADE. It was reported that biomass productivity, lipid content and nutrition removal efficiency were all dependent on the dilution ratio [25]. The ADE concentration reported in the literature generally varies between 2 and 50%, depending on species of microalgae, sources of ADE and operation of cultivation. Deionized water [24], seawater [26], tap water and normal medium [27] have been used as diluent, but biomass productivities were lower than those reported with pure synthetic media. Therefore, searching for a cost-effective diluent is important for large-scale microalgae cultivation. Municipal wastewater is better choice due to containing some nutrition such as N and P, which were essential elements for microalgal cell growth. The cultivation of microalgae could be achieved by simultaneously harvesting biomass, lipid accumulation and nutrition removal from anaerobic digested effluent from cattle manure (ADEC) combined with municipal wastewater. However, limited researches about above mentioned idea were reported. In a study, municipal wastewater with low concentration of nutrients was used as diluent, which can reduce the need for filtration and provide non-drinking water sources for dilution [28]. And the enhancement of ADE and municipal wastewater in microalgal growth was found to be wastewater- and strain dependent [28, 29]. Further, ADE and municipal wastewater contain various bacteria, posing contamination risks. Nutrients deficiency and unbalance in diluted ADE usually brought long cultivation period and poor biomass production [30]. Thus, it is necessary to investigate the sterilization and nutrient supply in diluted ADE.

The objectives of present study are to (1) investigate the feasibility of anaerobic digested effluent from cattle manure (ADEC) combined with municipal wastewater to cultivate microalgae; (2) harvest high biomass of microalgae based on ADEC medium. The pretreatment containing ADEC concentration, nutrient additives, diluents and sterilization operation was investigated to enhance the adaptability of microalgae in the ADEC medium. And then nutrients composite of ADEC medium, initial pH and light intensity were optimized for further enhancing the algal biomass accumulation.

## Results and discussion

### Effects of ADEC concentration and nutrient additives on microalgae growth in ADEC basal medium

**Fig. 1:**
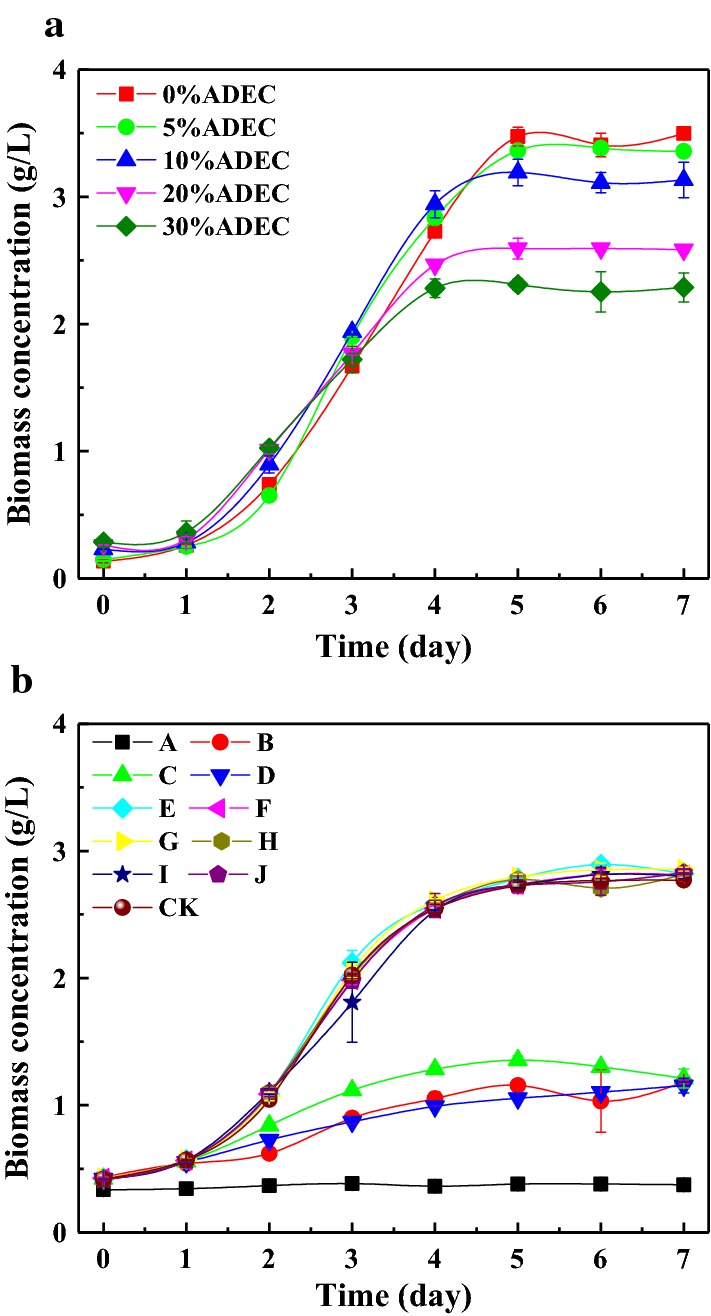
Effects of ADEC concentration (**a**) and nutrient-lacking (**b**) on biomass concentration of *Scenedesmus* sp. Mean and standard deviation of duplicate are shown

To explore the tolerance ability of *Scenedesmus* sp. L-1 to ADEC concentration, the ADEC was diluted with modified BG-11 medium into 0–30% of ADEC. The exponential growth period of microalgae in 0%, 5% and 10% ADEC basal medium lasted for 4 days, which was 1 day longer than that in 20% and 30% ADEC basal medium (Fig. 1a). Biomass concentration decreased gradually with the increase of ADEC concentration in ADEC basal medium. The biomass concentration of *Scenedesmus* sp. cultured in BG-11 medium was 3.50 g/L, while that in the medium containing 30% of ADEC was 2.29 g/L. This observation can be largely attributed to nutrients differences resulted from dilution ratio with modified BG-11 medium, which led to the inhibition of high concentration indicating that ADEC may not completely replace the nutrients in modified BG-11 medium. In addition, higher turbidity and dark color of dense ADEC in high concentration may hinder light penetration, which subsequently hampers growth of microalgae due to light limitation [17, 31]. As suggested by Markou et al. [32], ammonia had multiple impacts on the photosynthetic apparatus; photosystems I and II, the electron transport chain, the oxygen-evolution complex as well as the dark respiration were gradually inhibited by increasing ammonia nitrogen concentration. Excessive concentration of ammonia nitrogen to be tolerated by microalgae could be another explanation for growth inhibition [25]. Among them, biomass concentration of 5% and 10% ADEC medium in 7 days reached 3.36 and 3.13 g/L, respectively. Compared to other studies without nutrients addition like Ref. [33] (1.039 g/L after 40-day fed-batch cultivation of *Desmodesmus* sp. in 5% ADE) and Ref. [17] (1.57 g/L after 21-day cultivation of *Chlorella* sp. in 10% ADE), and ignoring the difference of operational conditions of the current experiment and the literature, the result of this work indicated that nutrient additives can shorten cultivation time and enhance biomass production. As reported by Franchino et al. [34], 1:10 digestate dilution ratio did not limit the growth of *Neochloris oleoabundans*, *Chlorella vulgaris* and *Scenedesmus obliquus*, allowing treatment of larger volumes of digestate with a higher initial nutrient concentration. To increase the treatment capacity of ADEC and reduce the use of diluent, it is better to choose 10% ADEC as the optimal ADEC concentration in basal medium for further studies.

To reduce the cost of cultivation in nutrients adding, it is necessary to supplement certain essential nutrients for microalgae growth instead of adding all nutrients of modified BG-11 medium. In the basal medium containing 10% ADEC, ten kinds of nutritional deficiencies were set up according to modified BG-11 medium. Figure 1b showed that the growth curve of Group E-J shared the similar growth trend with CK, and the biomass concentration was also similar to that in CK (2.77 g/L). This indicated that these nutrients including CaCl_2_, Na_2_CO_3_, citric acid, ammonium ferric citrate, EDTA-2Na and trace element A5 solution were non-essential for microalgae growth. However, no obvious growth was observed in Group A (lacking glucose). And the biomass concentration without glucose addition was merely 0.37 g/L. The growth of microalgae in Groups B, C and D (lacking NaNO_3_, K_2_HPO_4_·3H_2_O and MgSO_4_·7H_2_O) was inhibited, with the biomass concentration 1.18, 1.21 and 1.15 g/L, respectively. This indicated that ADEC can be used as a substitute for nutrient complete medium in BG-11 medium except carbon, similar with anaerobically digested piggery wastewater for MSE medium [27]. Therefore, glucose, NaNO_3_, K_2_HPO_4_·3H_2_O and MgSO_4_·7H_2_O were selected as essential nutrient additives in ADEC basal medium. Other non-essential nutrient additives were further determined by orthogonal experiments (Table 1). Those factors in descending order of biomass productivity: trace element A5 solution > EDTA-2Na > CaCl_2_ = Na_2_CO_3_ > Citric acid > Ammonium ferric citrate. The addition of both citric acid and trace elements enhanced biomass productivity. Ammonium ferric citrate had little effect on the biomass productivity. Therefore, the nutrient additives of ADEC basal medium were determined as glucose, NaNO_3_, K_2_HPO_4_·3H_2_O, MgSO_4_·7H_2_O, citric acid and trace element A5 solution.

**Table 1:** Results of non-essential nutrients addition orthogonal experiment

Group number	CaCl_2_	Na_2_CO_3_	Citric acid	Ammonium ferric citrate	EDTA-2Na	Trace elements	Biomass productivity (mg/L/day)
1	−	−	−	+	+	−	368.57
2	+	−	+	−	+	−	374.29
3	+	+	−	+	−	−	382.86
4	−	−	+	+	−	+	428.57
5	+	+	+	+	+	+	385.71
6	+	−	−	−	−	+	405.71
7	−	+	+	−	−	−	385.71
8	−	+	−	−	+	+	394.29
*K*_+_	387.14	387.14	393.57	391.43	380.71	403.57	
*K*_−_	394.29	394.29	387.86	390.00	400.71	377.86	
*R*	7.14	7.14	5.71	1.43	20.00	25.71	

“+” means be added, “−” means not be added

“*K*_*i*_” means average value of examination target at level i of each factor (*i* =+, −). “*R*” means the range of *K*_*i*_ of each factor

### Effects of dilution ratio and sterilization on microalgae growth and lipid production in ADEC

**Fig. 2:**
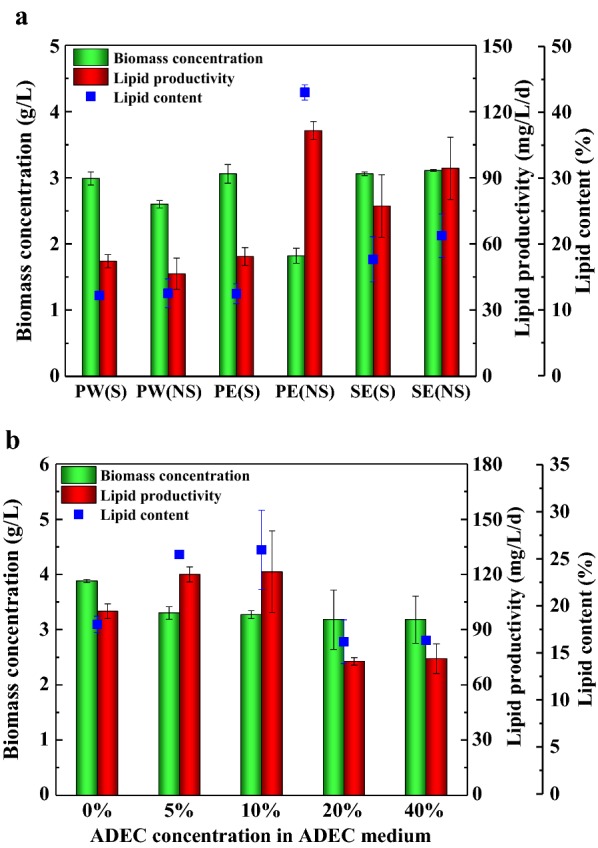
Effects of diluents and sterilization (**a**) and ADEC concentration in ADEC medium (**b**) on biomass concentration, lipid productivity and lipid content of *Scenedesmus* sp. Mean and standard deviation of duplicate are shown

PW, PE, and SE with or without sterilization were carried out to investigate their effects on the cell growth and lipid production of microalgae. Results showed that biomass concentration of SE(S) and SE(NS) reached 3.06 and 3.11 g/L, which were similar to that of PW(S) and PE(S) (Fig. 2a). The biomass concentration of PE(S) and PW(S) was higher than that of PE(NS) and PW(NS), respectively. The biomass concentration, lipid productivity and lipid content of PE(NS) were 1.82 g/L, 111.43 mg/L/d and 43%, respectively. It was reported that PE has higher bacterial contamination than SE, which may be harmful to the growth of microalgae and compete for organic carbon and other nutrients, making a stress to stimulate lipid accumulation [29]. Except from PE(NS), the lipid content in SE(NS) was up to 21%, followed by 18% in SE(S), 13% in PW, and 12% in both PE(S) and PW(S). Overall, both PE(NS) and SE(NS) were suitable as diluents in ADEC medium for lipid production. Since the biomass productivity of PE(NS) was too low and unstable, SE(NS) was chosen to increase the operability of the ADEC medium.

To verify the effect of ADEC concentration on microalgal growth and lipid production in ADEC medium diluted with SE without sterilization, the microalgae were cultivated in 0–40% (*v*_ADEC_/*v*_Medium_) of ADEC medium. From Fig. 2b, the biomass concentration in 5–40% ADEC medium (3.18–3.30 g/L) was lower than that in 0% ADEC (3.88 g/L). Different from the results of BG-11 medium as diluent before, there was little difference of biomass concentration in range of 5–40% ADEC. This indicated that under the same nutrient addition conditions, the difference in final nutrient level, color and inhibitor content caused by ADEC concentration might not affect the growth of *Scenedesmus* sp. With the ADEC concentration increasing from 0 to 10%, the lipid productivity and lipid content increased from 100.00 mg/L/day and 18% to 121.43 mg/L/day and 26%, respectively. In higher concentration of 20–40%, both lipid productivity and content decreased and maintained at 72.86–74.29 mg/L/day and 16.23–16.38%, respectively. The effect of ADEC concentration on lipid production behaved differently, the reasons of which were mainly due to concentration of organic carbon and dense of color in wastewaters. The content of organic carbon increased with the ADEC concentration. As we know, organic carbon benefited lipid accumulation; that is why lipid productivity and lipid content increased in lower ADEC concentration. As to the dense of color, the color deepened with the concentration of ADEC. Maybe in lower ADEC concentration of 0–10% (v/v), the wastewaters were in range of colorless to light brown, which had less effect on lipid accumulation compared to the effect of organic carbon. In higher concentration of 20–40% (v/v), the dense of color got dark and reduced the light penetration severely, thus affecting photosynthesis and lipid production of microalgae. To sum up, SE with non-sterilization was used as diluent, and 10% was chosen as the optimal concentration of ADEC in the ADEC medium.

### Effect of essential nutrients concentration on microalgae growth and lipid production in ADEC medium

**Fig. 3:**
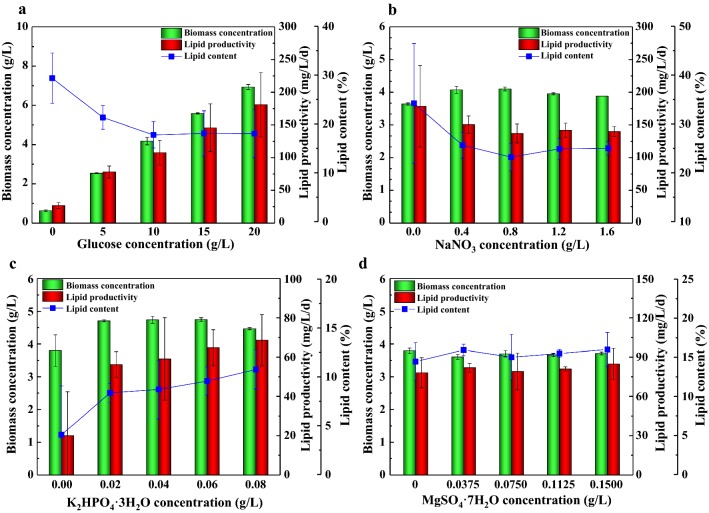
Effects of glucose (**a**), NaNO_3_ (**b**), K_2_HPO_4_·3H_2_O (**c**), MgSO_4_·7H_2_O (**d**) concentrations on biomass concentration, lipid productivity and lipid content of *Scenedesmus* sp. in ADEC medium. Mean and standard deviation of triplicate are shown

To determine the best nutrient additives, the influence of four essential nutrients (glucose, NaNO_3_, K_2_HPO_4_·3H_2_O, MgSO_4_·7H_2_O on the cell growth and lipid production of *Scenedesmus* sp. was investigated (Fig. 3). With the increase of glucose concentration, the biomass concentration and lipid productivity increased gradually and the maximum biomass concentration of 6.93 g/L and maximum lipid productivity of 180.95 mg/L/day with 20 g/L of glucose addition was achieved (Fig. 3a). However, the lipid content decreased from 30% without glucose addition, and leveled out at 18% with 10 g/L of glucose, where the biomass concentration and lipid productivity were 4.18 g/L and 107.62 mg/L/day, respectively. The results indicated that higher concentration of glucose did not increase the lipid content of microalgae, which was similar to the report of Mandal and Mallick [35]. Ren et al. also found glucose (10 g/L) as the best carbon source for *Scenedesmus* sp. [21]. Therefore, based on the actual effect on biomass and lipid production, and considering of substrate saving, the optimum concentration of glucose was determined to be 10 g/L. However, there existed some contention of glucose for it is an expensive substrate, which could not be sustainable large-up culture. Thus, future studies focusing on seeking for cheaper carbon source such as molasses wastewater for mixotrophic cultivation would be imperative [36].

Nitrogen is an essential nutrient for the growth and lipid synthesis of microalgae [37]. Although nitrogen starvation could induce lipid accumulation, abundant nitrogen sources are more conducive to maintaining higher growth rate and achieving higher biomass concentration of microalgae. Thus, five various NaNO_3_ concentrations (0, 0.4, 0.8, 1.2 and 1.6 g/L) were designed to investigate their effect on cell growth and lipid accumulation of *Scenedesmus* sp. (Fig. 3b). Maximum biomass concentration was obtained at 0.8 g/L of nitrogen, up to 4.10 g/L. Comparable result (4.07 g/L) could be achieved at 0.4 g/L of nitrogen. The control without nitrogen addition showed the maximum lipid productivity (178.57 mg/L/day) and lipid content (34%). On the contrary, the algal growth performed poor without nitrogen addition. The color of medium changed from light green to yellow during the entire culture time. This change in color may be that nitrogen deficiency resulted in the abnormal synthesis or degradation of chlorophyll to provide nitrogen sources for cell growth [38]. Certain reports showed that microalgae cells accumulated large quantities of chlorophyll when nitrogen source was abundant, while utilized chlorophylls as a nitrogen source when nutrients were exhausted [39, 40]. Especially, chlorophyll content reduced after 48 h of nitrogen starvation. Compared to other treatments, N starvation led to significant increases in chlorophyll a on day 1, followed by significant decrease on day 2 and an overall decrease in chlorophyll content (a and b) on day 3 [41]. Since the effect of nitrate concentration on chlorophyll content has been elaborated in most previous studies, the value of chlorophyll content was not incorporated. Thus, the optimum concentration of NaNO_3_ was determined to be 0.4 g/L in this experiment.

Phosphorus is an essential element in DNA, RNA, ATP and cell membrane, and plays an important role in signal transduction and cell metabolism [42]. Phosphorus deficiency leads to the decrease of enzyme activity and the limitation of NADH and ATP synthesis in Calvin cycle. Therefore, low concentration of phosphorus could affect the cell division and chlorophyll synthesis, as well as the fatty acid metabolism, resulting in lower biomass and lipid production [43]. When K_2_HPO_4_·3H_2_O was not added, the cell growth of *Scenedesmus* sp. was limited, and its biomass at stationary phase was 3.80 g/L, similar to the lipid productivity (20.00 mg/L/day) and lipid content (4%), which were much lower than that of other experimental groups (Fig. 3c). Biomass concentration of 4.71–4.75 g/L was obtained with the addition of 0.02–0.06 g/L K_2_HPO_4_·3H_2_O. A slight decrease in biomass concentration (4.47 g/L) occurred when higher concentration of K_2_HPO_4_·3H_2_O (0.08 g/L) was added. This inhibition of high phosphate on cell growth is in accordance with the previous study of *Chlorella* sp. performed by Liang et al. [44]. With the increase of K_2_HPO_4_·3H_2_O concentration from 0.02 to 0.08 g/L, the lipid productivity increased slowly from 56.19 to 68.57 mg/L/day, while the lipid content increased from 8 to 11%. Thus, the optimum concentration of K_2_HPO_4_·3H_2_O was determined at 0.02 g/L.

Magnesium is an essential constituent of the chlorophyll molecule and macronutrient for algal growth. Besides, Mg^2+^ could promote the activity of acetyl coenzyme A carboxylase (the key enzyme for fatty acid synthesis) and increase the content of neutral lipid in microalgae cells [45]. According to Fig. 3d, adding different concentrations of MgSO_4_·7H_2_O (0–0.150 g/L) had no distinct effect on the growth and lipid production of *Scenedesmus* sp. The biomass concentration and lipid productivity leveled off at 3.70 g/L and 80.95 mg/L/day, and the lipid content fluctuated at 15%. MgSO_4_ was not an essential nutrient additive in the ADEC medium, different with the result in ADEC basal medium. Maybe SE as diluent could supplement some Mg^2+^. Similar with McGinn et al. [46], they suggested that there would likely be no need to add trace elements such as Mg^2+^ to achieve high biomass and efficient nutrient drawdown when AD wastes were diluted by municipal wastewater. However, it has been reported that the anaerobic digestion of effluent from cow manure and supplementation of magnesium have improved the productivity of *Scenedesmus* sp. AMDD in culture medium [47]. Considering those controversial views, MgSO_4_·7H_2_O was used as a non-essential nutrient additive in further orthogonal experiment of nutritional composition.

Six nutrients such as glucose, NaNO_3_, K_2_HPO_4_·3H_2_O, MgSO_4_·7H_2_O, citric acid and trace element A5 solution were designed by an orthogonal experiment (Table 2). According to the results, six factors affecting the growth of microalgae were in declined order: glucose > K_2_HPO_4_·3H_2_O > citric acid > NaNO_3_ > MgSO_4_·7H_2_O > trace element A5 solution. The optimum nutrient composition for growth in the ADEC medium was: glucose 10 g/L, NaNO_3_ 0.3 g/L, K_2_HPO_4_·3H_2_O 0.01 g/L, MgSO_4_·7H_2_O 0.075 g/L and trace element A5 solution 1 mL/L.

**Table 2:** The orthogonal experimental results of nutrient composite in ADEC medium

Number	Glucose (g/L)	NaNO_3_ (g/L)	K_2_HPO_4_·3H_2_O (g/L)	MgSO_4_·7H_2_O (g/L)	Citric acid (g/L)	Trace elements (mL/L)	Biomass concentration (g/L)
1	10	0.3	0.010	0	0	0	4.08
2	6	0.4	0.010	0	0	0	2.42
3	10	0.2	0.020	0	0.006	0	3.74
4	6	0.2	0.010	0.075	0.006	1	2.44
5	8	0.2	0.010	0	0	1	3.52
6	8	0.3	0.010	0.075	0.006	0	3.36
7	6	0.4	0.010	0.075	0.006	0	2.38
8	6	0.2	0.010	0	0	1	2.64
9	10	0.2	0.010	0.075	0.006	1	4.04
10	8	0.4	0.020	0	0.006	1	2.80
11	6	0.3	0.015	0	0.006	1	2.44
12	8	0.2	0.015	0.075	0	0	3.26
13	6	0.2	0.020	0.075	0	0	2.48
14	6	0.2	0.015	0	0.006	0	2.48
15	10	0.4	0.015	0.075	0	1	4.14
16	6	0.3	0.020	0.075	0	1	2.48
Biomass concentration							
*K*_1_	2.438	3.244	3.291	3.174	3.286	3.184	
*K*_2_	3.203	3.259	3.261	3.231	3.119	3.221	
*K*_3_	3.968	3.104	3.056				
*R*	1.530	0.155	0.235	0.057	0.168	0.037	

“*K*_*i*_” means average value of examination target at level *i* of each factor (*i* = 1, 2, 3). “*R*” means the range of *K*_*i*_ of each factor

### The growth, lipid production and nutrients utilization in ADEC growth medium

**Fig. 4:**
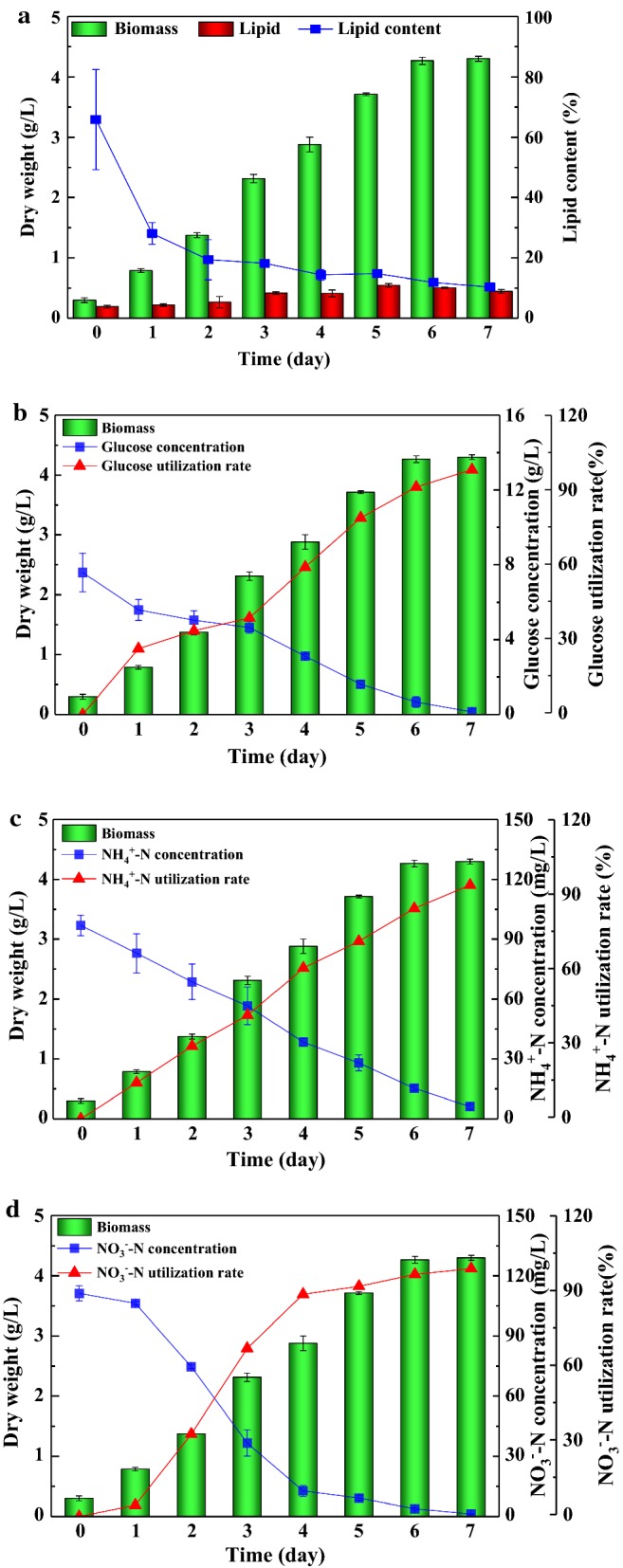
The biomass, lipid production (**a**) and nutrients removal efficiency (**b**–**e**) of *Scenedesmus* sp. in BG-11 and ADEC growth medium. Mean and standard deviation of triplicate are shown

As shown in Fig. 4, a time-course of biomass concentration and lipid production, and nutrients utilization of *Scenedesmus* sp. in the ADEC growth medium was exhibited. The microalgae adapted to the ADEC growth medium quickly, with no obvious lag phase, suggesting that this species was suitable to ADEC wastewater with fast growth. Maximum biomass concentration was achieved at 4.30 g/L in 7 days, with the utilization rate of glucose, ammonia nitrogen and nitrogen reaching to 98.16%, 93.68% and 98.89%, respectively. The maximum lipid yield (0.55 g/L) was obtained on 5th day, which may partly due to the increase of biomass concentration and nitrogen depletion stimulating lipid accumulation in the culture process. However, lipid content decreased sharply over time, and finally stabilized at 10.39%. The results indicated that there was a natural contradiction between biomass and lipid content in the process of synergistic growth and lipid production [48]. A further study of two-stage cultivation with a high-density culture stage and lipid-inducing stage separately may be a solution to enhance lipid productivity [49].

Interestingly, the utilization rate of NH_4_^+^-N by *Scenedesmus* sp. went straight up over 7 days. The decrease of ammonia nitrogen concentration is mainly due to the absorption and utilization of microalgae, as well as bacterial uptake in wastewater medium and surface adsorption of microalgae. The initial concentration of NH_4_^+^-N in ADEC medium was approximately 100 mg/L, which had no inhibitory or toxic effect on the growth of *Scenedesmus* sp. L-1. A similar phenomenon was observed of *Scenedesmus accuminatus* in ADE by Park et al. [50]. However, the effect of high ammonia nitrogen concentration was species specific. The same concentration of 100 mg/L NH_4_^+^-N was reported to be toxic to *Neochloris oleoabundans* [24]. The characteristics of NO_3_^−^-N utilization seemed to be related to the growth of *Scenedesmus* sp. L-1. The utilization rate of NO_3_^−^-N increased slowly on 1st day. This could be explained by that microalgae preferentially use NH_4_^+^-N when NH_4_^+^-N and NO_3_^−^-N coexist in wastewater. The utilization rate of NO_3_^−^-N increased sharply in exponential growth period, and the utilization rate of NO_3_^−^-N reached 88.60% in 4 days. NO_3_^−^-N was absorbed and stored in intracellular nitrogen pool to support growth and lipid production. Even though the nitrogen concentration reduced to stimulate lipid accumulation, the glucose exhaustion may lead to a decrease in lipid productivity in the late culture period [20]. In other words, the availability of glucose appears to play a major role in lipid accumulation. Similar with anaerobically digested dairy manure [24], the ADEC medium showed relatively stable pH (7.0–7.6) over experimental period, indicating that it had a good buffer effect and no additional regulation of pH was needed during the culture period.

According to the results, ADEC growth medium consisted in ADEC and secondary effluent of municipal wastewater with certain essential nutrient additives without sterilization. The possibility of bacterial infection needs to be discussed. Compared to PE(NS), SE(NS) has lower bacterial contamination, proved by the results of microalgal growth in mediums of various diluents and sterilization. In addition, the microalgae seeds were pure culture and incubated in ADEC growth medium in super-clean worktable, and then cultivated in a batch culture in incubator. Those operations could reduce the possibility of bacterial infection. Thus, there is less bacterial infection of microalgae cultivated in ADEC growth medium.

### Effect of initial pH on biomass, lipid production and wastewater treatment in ADEC growth medium

**Fig. 5:**
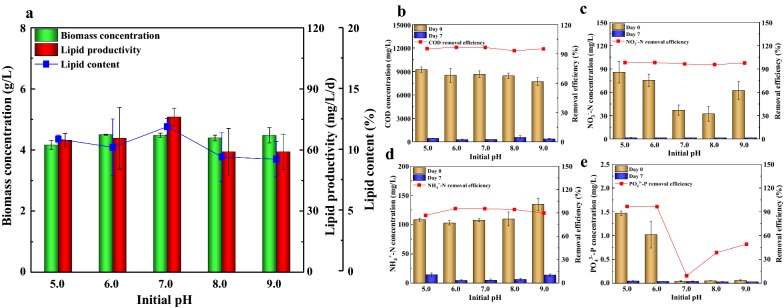
Effect of initial pH values on the growth, lipid production (**a**) and nutrients removal efficiency (**b**–**e**) of *Scenedesmus* sp. in ADEC growth medium. Mean and standard deviation of triplicate are shown

Initial pH has a significant effect on cell surface properties, which can change the biochemical metabolism and enzyme system activity of microalgae [51], and thus the effects of initial pH on growth and lipid production of *Scenedesmus* sp. in ADEC growth medium were studied. Among the test initial pH ranged from 5.0 to 9.0, initial pH of 6.0 was best to achieve the maximum biomass concentration of 4.5 g/L (Fig. 5a). Comparable biomass concentration was exhibited when *Scenedesmus* sp. was cultured at pH 7.0–9.0. The adaptability of alga to grow on alkaline condition is supposed to increase its potential for large-scale outdoor conditions by inhibiting biological contaminants at higher pH range [52]. In another study with *Scenedesmus obliquus* M2-1, the biomass production was highest at pH 6.0 [53]. These microalgae apparently prefer a relatively low pH value. The maximum lipid productivity of 76.19 mg/L/day and maximum lipid content of 12% were obtained at pH 7.0. Similar to Breuer et al. [51], pH 7.0 was the optima for TAG accumulation in *Scenedesmus obliquus*. Thus, initial pH of 7.0 was chosen as the optimum pH of the ADEC growth medium for microalgal growth and lipid production.

Over 90% of COD, NO_3_^−^-N was removed at all tested initial pH values. The removal efficiency of NH_4_^+^-N performed better when initial pH was 6.0–8.0. The initial pH value may affect the distribution of PO_4_^3−^-P in ADEC medium. The initial concentration of PO_4_^3−^-P was higher under weak acid condition (initial pH = 5.0–6.0), with 1.47 mg/L and 1.02 mg/L, respectively. While it decreased significantly under neutral and weak alkali conditions (initial pH = 7.0, 8.0 and 9.0), which was about 0.05 mg/L. The decrease of PO_4_^3−^-P concentration in ADE was due to not only the absorption and utilization of microalgae, surface adsorption, but also the precipitation of phosphorus by calcium and magnesium [25]. When the pH value is between 8.0 and 11.0, insoluble precipitates (i.e. hydroxyapatite and struvite) are easily produced in the presence of ions such as Ca^2+^, PO_4_^3−^, NH_4_^+^ and Mg^2+^ [54]. Although different initial pH values resulted in distinct differences in initial PO_4_^3−^-P concentrations, acceptable biomass and lipid accumulation were achieved in all experimental groups. At the end of culture, the residual PO_4_^3−^-P concentration in each experimental group decreased to 0.03–0.05 mg/L.

### Effect of light intensity on biomass, lipid production and wastewater treatment in ADEC growth medium

**Fig. 6:**
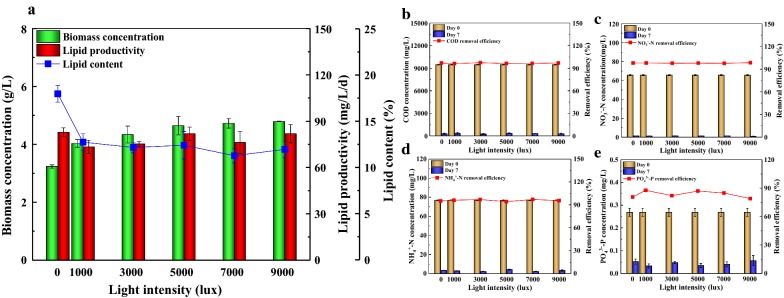
Effect of light intensity on the growth, lipid production (**a**) and nutrients removal efficiency (**b**–**e**) of *Scenedesmus* sp. in ADEC growth medium. Mean and standard deviation of triplicate are shown

Light intensity is a key factor affecting cell metabolism such as growth, lipid synthesis and CO_2_ fixation of microalgae [55]. To investigate the best light intensity for the growth and lipid accumulation of *Scenedesmus* sp. on the ADEC growth medium, the light intensity of 0–9000 lx was studied. The effects of light intensity on the microalgal growth can be divided into three stages: light limitation, light saturation and high light inhibition. At a lower light intensity, biomass concentration of microalgae increased from 3.23 to 4.65 g/L with increasing light intensity from 0 to 5000 lx. However, no obvious increase of biomass concentration was observed with higher light intensity at 7000 and 9000 lx (Fig. 6a), suggesting the saturation point of photosynthesis was reached. The maximum lipid productivity of 81.90 mg/L/day was obtained at 5000 and 9000 lx. Overall, lipid productivity of *Scenedesmus* sp. fluctuated around 76.19 mg/L/day under different light intensity, indicating the lipid production could be independent of light intensity. Similar phenomenon was also reported by *Scenedesmus obliquus* [51]. As proposed by Simionato et al. [56], lipid accumulation is activated by nutrients limitation during the stationary phase when microalgae cells are exposed to a constant low or moderate light irradiation. Interestingly, high light stress has small effect on lipid production, which is induced by nutrients deprivation again, since cells response with enhancing protective mechanisms and optimizing their photosynthetic apparatus. Under dark condition, the lipid content was as high as 18%, while the lipid content corresponding to 1000–9000 lx was about 12%. Ho et al. demonstrated that the lipid productivity of *Scenedesmus obliquus* CNW-N was highly related to biomass productivity since no clear variation was observed on the lipid content of this strain [55]. It is worth underlining that this capacity of maintaining the constant lipid productivity in a wide range of light conditions is a valuable property in using this species for biofuels production outdoor with variable light intensity. The removal efficiency of COD, NO_3_^−^-N and NH_4_^+^-N reached more than 90% at all tested light intensity, and the residual concentration were 282–353 mg/L, 0.91–1.36 mg/L, and 2.2–4.4 mg/L, respectively. 79–88% of PO_4_^3−^-P was removed, remaining 0.03–0.06 mg/L at the end of the cultivation. Considering about energy income, 5000 lx would be better as the optimum light intensity for the cell growth of *Scenedesmus* sp.

### Comparison of microalgae cultivated in ADEC growth medium and modified BG-11 medium

To further understand the performance of microalgae cultivated in ADEC growth medium, the comparison of growth, lipid production and fatty acid methyl esters (FAMEs) in ADEC growth medium and modified BG-11 medium was investigated. According to Table 3, the biomass concentration of 4.82 g/L was obtained in ADCE growth medium, 12.62% higher than that in modified BG-11 medium. On the contrary, the lipid content of 15.25% was recorded in ADEC growth medium, 8.79% lower than that in modified growth medium. This indicated that the contradiction of microalgal growth and lipid content existed in ADEC growth medium as in modified BG-11 medium. As a result, the lipid productivity in ADEC growth medium and modified BG-11 medium shared the similar values, recorded as 104.76 and 102.38 mg/L/day, respectively. As to FAME profile of *Scenedesmus* sp., the main fatty acids were in range of C14:0 to C18:0 in two kinds of mediums. As we know, C16- to C18-chain-length fatty acids were the most required for biodiesel production [57]. In this study, C16–C18 FAs accounted for 98.22% of total FAME composition in ADEC growth medium, similar to that for 98.02% in modified BG-11 medium. Among them, oleic acid (C18:1 w9c) is the predominant fatty acid with the range of 43.41% followed by palmitic acid (C16:0) with 25.88% in ADEC growth medium, both higher than that in modified BG-11 medium. Another quality of biodiesel is low temperature performance, which is affected by the saturation degree of fatty acids. Usually, higher saturated FAs makes the melting point of biodiesel higher, while higher unsaturated fatty acid makes that lower. However, there existed a problem that higher unsaturated fatty acids were easily oxidized. Monounsaturated fatty acids had good oxidation stability and low temperature fluidity [58]. Therefore, the FAME profile obtained by ADEC growth medium could ensure the stability and low temperature performance of biodiesel due to its composition as saturated (39.48%) and monounsaturated (60.52%) FAs, similar to the composition as saturated (40.42%) and monounsaturated (59.69%) FAs of microalgae cultivated by modified BG-11 medium.

**Table 3:** The growth, lipid production and fatty acid methyl esters (FAME) of *Scenedesmus* sp. in modified BG-11 medium and ADEC growth medium

	Modified BG-11 medium		ADEC growth medium	
	Mean	SD	Mean	SD
Biomass concentration (g/L)	4.28	0.13	4.82	0.24
Lipid content (%)	16.72	0.82	15.25	1.74
Lipid productivity (mg/L/day)	102.38	7.43	104.76	10.31
C14:0	0.34	0.01	0.43	0.03
C15:0 3OH	1.69	0.51	1.35	0.12
C16:0	22.14	1.12	25.88	0.57
C16:0 iso	14.56	1.86	8.78	1.00
C16:1 w11c	19.72	0.90	17.11	0.80
C18:0	1.69	0.18	3.04	0.14
C18:1 w9c	39.97	1.06	43.41	1.02
C16–18 FAs	98.08		98.22	
Saturated FAs	40.42		39.48	
Monounsaturated FAs	59.69		60.52	

C16–18 FAs = C16:0 + C16:0 iso + C16:1 w11c + C18:0 + C18:1 w9c

Saturated FAs = C14:0 + C15:0 3OH + C16:0 + C16:0 iso + C18:0

Monounsaturated FAs = C16:1 w11c + C18:1 w9c

## Conclusions

This study demonstrated that *Scenedesmus* sp. could grow rapidly in 10% anaerobic digestion of cattle manure effluent with secondary effluent as diluents without sterilization. Nutrient additives for fast growth of microalgae are necessary. The biomass concentration of 4.65 g/L and lipid productivity of 81.9 mg/L/day during 7-day cultivation were achieved in optimized culture condition with pH of 7.0 and light intensity of 5000 lx, respectively. The removal efficiency of COD, NO_3_^−^-N and NH_4_^+^-N were more than 90%. The FAME profile in ADEC growth medium consisted in saturated (39.48%) and monounsaturated (60.52%) fatty acids with higher proportion of oleic acid and palmitic acid. And the 16- to 18-chain-length fatty acids accounted for more than 98% of total FAMEs. These lipid properties make sure the stability and low temperature performance of biodiesel. These results showed that *Scenedesmus* sp. could potentially grow and accumulate lipids in ADEC coupling with wastewater treatment.

## Methods

### Microalgal species and culture conditions

The microalgae *Scenedesmus* sp. strain L-1 preserved in the Laboratory of Environment and Biotechnology in Harbin Institute of Technology was applied in this study for its advantages in fast growth and nitrogen and phosphorus removal efficiency. BG-11 medium has universal nutrients suitable for the growth of *Scenedesmus* sp. (green algae), and BG-11 medium was also commonly used in the culture of *Scenedesmus* sp. in the literature [28, 59, 60]; so BG-11 medium was chosen in this study. BG-11 medium consisted in NaNO_3_ 0.8 g/L, K_2_HPO_4_·3H_2_O 0.04 g/L, MgSO_4_·7H_2_O 0.075 g/L, CaCl_2_ 0.027 g/L, Na_2_CO_3_ 0.02 g/L, citric acid 0.006 g/L, ammonium ferric citrate 0.006 g/L, EDTA-2Na 0.001 g/L, and trace element A5 solution 1 mL/L. A5 is a trace metal solution containing H_3_BO_3_ 2.86 g/L, MnCl_2_·4H_2_O 1.81 g/L, ZnSO_4_·7H_2_O 0.22 g/L, Na_2_MoO_4_·2H_2_O 0.39 g/L, CuSO_4_·5H_2_O 0.08 g/L, and Co(NO_3_)_2_·6H_2_O 0.04 g/L. The culture medium was supplemented with 10 g/L glucose as modified BG-11 medium. The initial pH was adjusted to 7.0. All the experiments were conducted in 250-mL Erlenmeyer flasks containing 150-mL autoclaved modified BG-11 medium with 10% (v/v) inoculation and were incubated in a shaker under white fluorescent light (2500 lx with a light/dark cycles of 12 h/12 h) at 25 °C in batch culture [61].

### Wastewaters

The anaerobic digested effluent from cattle manure (ADEC) was collected from Northeast Agricultural University in Harbin, China. The primary sedimentation tank effluent (PE) and secondary sedimentation tank effluent (SE) were obtained from Wenchang Sewage Treatment Plant in Harbin, China. The supernatant of all the wastewater were collected after static treatment for 1 week and ADEC with a further centrifuge at 8000 rpm for 3 min to remove large particles. The chemical oxygen demand (COD), nitrate (NO_3_^−^-N), ammonia (NH_4_^+^-N) and phosphorus (PO_4_^3−^-P) concentration in ADEC, PE and SE were shown in Table 4. The ADEC basals medium consisting in ADEC and modified BG-11 medium was used after sterilization. In experiment of wastewater for microalgal culture, the seeds were inoculated in the mixture of ADEC and pure water (PW), or ADEC and PE, or ADEC and SE, with or without sterilization.

**Table 4:** The characteristic of ADEC, PE and SE

	ADEC		PE		SE	
	Mean	SD	Mean	SD	Mean	SD
NH_4_^+^-N (mg/L)	424.45	23.12	41.53	5.18	0	0
NO_3_^−^-N (mg/L)	17.86	1.84	0.50	0.10	5.93	1.03
PO_4_^3−^-P (mg/L)	18.31	0.12	4.49	0.06	0.09	0.00
COD (mg/L)	2654	236	52	22	24	20

### The pretreatment of ADEC

To determine the optimal ADEC concentration, ADEC was diluted with modified BG-11 into 0, 5, 10, 20 and 30% (*v*_ADEC_/*v*_Medium_). Then, the nutrient additives in ADEC basal medium containing 10% ADEC were screened according to a nutrients-lacking experiment (Table 5) and an orthogonal experiment of non-essential nutrients addition (Table 1) to determine the best combination of nutrient additives. PW, PE and SE were used as diluents with or without sterilization to make out the best diluent and sterilization. To verify the effect of pretreatment, the ADEC was diluted with municipal wastewater into 0, 5, 10, 20 and 40% (*v*_ADEC_/*v*_Medium_).

**Table 5:** The design of nutrients-lacking groups in nutrient-additives screening experiment

Nutrients	A	B	C	D	E	F	G	H	I	J	CK
Glucose	−	+	+	+	+	+	+	+	+	+	+
NaNO_3_	+	−	+	+	+	+	+	+	+	+	+
K_2_HPO_4_·3H_2_O	+	+	−	+	+	+	+	+	+	+	+
MgSO_4_·7H_2_O	+	+	+	−	+	+	+	+	+	+	+
CaCl_2_	+	+	+	+	−	+	+	+	+	+	+
Na_2_CO_3_	+	+	+	+	+	−	+	+	+	+	+
Citric acid	+	+	+	+	+	+	−	+	+	+	+
Ammonia ferric citrate	+	+	+	+	+	+	+	−	+	+	+
EDTA-2Na	+	+	+	+	+	+	+	+	−	+	+
Trace element	+	+	+	+	+	+	+	+	+	−	+

“+”means be added, “−” means not be added

### The optimization of nutrients additives in ADEC medium and microalgal culture conditions

To further enhance the growth of *Scenedesmus* sp., the effect of glucose, nitrogen, phosphorus and magnesium supply was studied firstly, and the optimum range of nutrient concentration was determined by the biomass, lipid productivity and lipid content of microalgae. Orthogonal experiment (Table 2) was used to optimize the nutrient composition in ADEC medium. And then, a time-course observation in growth, lipid production and nutrient utilization was carried out in ADEC growth medium. The initial pH (5.0, 6.0, 7.0, 8.0 and 9.0) and light intensity (0, 1000, 3000, 5000, 7000 and 9000 lx) were optimized finally.

### The comparison of microalgae cultivated in ADEC growth medium and modified BG-11 medium

To verify the performance of microalgae cultured in ADEC growth medium under the optimized initial pH and light intensity, the biomass concentration, lipid production and fatty acids composition of microalgae in ADEC growth medium were compared to that in modified BG-11 medium.

### Analytical methods

Biomass concentration was determined by optical density measurements at 680 nm (OD_680_). The linear regression equation of biomass concentration and OD_680_ was: Biomass concentration = OD_680_ × 3.13, *R*^2^ = 0.9915. The method of biomass concentration on day 7 referred to Ma et al. [61]. Biomass was collected by centrifugation, followed by washing with 0.1 mmol/L PBS, and then weighed on an electronic scale after drying at 60 °C to constant weight. Lipid content was determined using Bligh and Dyer’s method with a slight modification [62]. Lipids were extracted from biomass with chloroform: methanol: pure water (1:2: 0.8, v/v/v) for an ultrasonic crush. Chloroform and pure water were added to give a final solvent ratio of chloroform: methanol: water solvent ratio of 1:1:0.9 (v/v/v). The supernatant recovered and the same process was carried out twice for the complete extraction of lipids. Biomass productivity and lipid productivity were calculated according to the following Eqs. (1) and (2). The analysis of fatty acid composition was performed by gas chromatography [63]. The concentration of COD, NO_3_^−^-N, NH_4_^+^-N and PO_4_^3−^-P were determined according to Yang et al. [64]. Glucose concentration in the medium was measured by phenol–sulfuric acid method [65]. All the samples were filtered (0.45 μm) to eliminate the influence of particles and microalgae biomass in measurements. The nutrient utilization rate and removal efficiency were calculated by the following Eqs. (3) and (4).

1 $${\text{Biomass}}\;{\text{productivity }}\left( {{\text{mg}}/{\text{L}}/{\text{day}}} \right) \, = \, (C_{t} - C_{0} )/t$$

Here, *C*_0_ and *C*_*t*_ were the biomass concentration in day 0 and day 7, respectively.

2 $${\text{Lipid productivity }}\left( {{\text{mg}}/{\text{L}}/{\text{day}}} \right) \, = {\text{ biomass concentration }} \times {\text{ lipid content}}/t$$

Here, *t* was the cultivation time (day), and equaled to 7 days in our experiments. This was due to the maximum biomass obtained on the 7th day.

3 $${\text{The utilization rate}}\,\left( \% \right) = \left( {C_{0} - \, C_{t} } \right)/ \, C_{0}$$

4 $${\text{The}}\;{\text{removal}}\;{\text{efficiency}}\,\left( \% \right) = \left( {C_{0} - \, C_{t} } \right)/ \, C_{0}$$

Here, *C*_0_ and *C*_*t*_ were the concentration in day 0 and 7, respectively.

## Data Availability

All data generated or analyzed during this study are included in this published article.

## Abbreviations

AD: anaerobic digestion
ADE: anaerobic digested effluent
ADEC: anaerobic digested effluent from cattle manure
EDTA-2Na: ethylenediaminetetraacetic acid disodium salt
C/N: carbon/nitrogen
NADH: nicotinamide adenine dinucleotide
ATP: adenosine triphosphate
TAG: triacylglycerol
COD: chemical oxygen demand
NO_3_^−^-N: nitrate
NH_4_^+^-N: ammonia
PO_4_^3^-P: phosphorus
PE: primary sedimentation tank effluent
SE: secondary sedimentation tank effluent
PW: pure water
FAMEs: fatty acid methyl esters
FAs: fatty acids
DNA: deoxyribonucleic acid
RNA: ribonucleic acid
